# Inflammation predicts new onset of depression in men, but not in women within a prospective, representative community cohort

**DOI:** 10.1038/s41598-021-81927-9

**Published:** 2021-01-26

**Authors:** Mareike Ernst, Elmar Brähler, Daniëlle Otten, Antonia M. Werner, Ana N. Tibubos, Iris Reiner, Felix Wicke, Jörg Wiltink, Matthias Michal, Markus Nagler, Thomas Münzel, Philipp S. Wild, Jochem König, Norbert Pfeiffer, Andreas Borta, Karl J. Lackner, Manfred E. Beutel

**Affiliations:** 1 Department of Psychosomatic Medicine and Psychotherapy, University Medical Center of the Johannes Gutenberg-University Mainz, Untere Zahlbacher Str. 8, 55131 Mainz, Germany; 2 Preventive Cardiology and Preventive Medicine – Department of Cardiology, University Medical Center of the Johannes Gutenberg-University Mainz, Mainz, Germany; 3 Center for Thrombosis and Hemostasis (CTH), University Medical Center of the Johannes Gutenberg-University Mainz, Mainz, Germany; 4 Department of Cardiology – Cardiology I, University Medical Center of the Johannes Gutenberg-University Mainz, Mainz, Germany; 5 Institute of Medical Biostatistics, Epidemiology and Informatics (IMBEI), University Medical Center of the Johannes Gutenberg-University Mainz, Mainz, Germany; 6 Department of Ophthalmology, University Medical Center of the Johannes Gutenberg-University Mainz, Mainz, Germany; 7 Boehringer Ingelheim Pharma GmbH Co KG, Ingelheim am Rhein, Germany; 8 Institute of Clinical Chemistry and Laboratory Medicine, University Medical Center of the Johannes Gutenberg-University Mainz, Mainz, Germany; 9 German Center for Cardiovascular Research (DZHK), Partner Site Rhine-Main, Mainz, Germany

**Keywords:** Human behaviour, Medical research, Depression, Biomarkers, Inflammation, Risk factors, Public health, Psychiatric disorders

## Abstract

Depression has been associated with increased inflammation. However, only few large-scale, prospective studies have evaluated whether inflammation leads to new cases of depression and whether this association can be found in men and women. Longitudinal data of *N* = 10,357 adult participants with no evidence of depression at baseline (based on Patient Health Questionnaire (PHQ-9), lifetime diagnoses, and current antidepressant medication) were evaluated for depression 5 years later. Multivariate logistic regression models were used to predict the onset of depression based on C-reactive protein (CRP) and white blood cell count (WBC). We used interaction terms and separate analyses in men and women to investigate gender-dependent associations. Based on both markers, inflammation was predictive of new cases of depression 5 years later, even when adjusting for sociodemographic, physical health, health behavior variables, and baseline depression symptoms. As established by interaction terms and separate analyses, inflammatory markers were predictive of depression in men, but not in women. Additional predictors of new onset of depression were younger age, loneliness, smoking (only in men), cancer and less alcohol consumption (only in women). The study indicates gender differences in the etiology of depressive disorders within the community, with a greater role of physical factors in men.

## Introduction

Depression has been recognized as one of the most frequent and harmful mental disorders with an estimated life time risk of 15–25%; with prevalence rates twice as high among women compared to men^1^. Researchers have discussed a variety of factors underlying this observation, for instance genetic and biological vulnerabilities, environmental factors such as daily stressors, and differences in emotion regulation and stress responsiveness^2^.

In recent years, a growing body of research examining the temporal dynamics of depressive disorders has identified close associations of depression symptoms with chronic physical diseases^3–5^. The association with cardiovascular disease (CVD), which caused 37% of deaths in the German population in 2017^6^, has been most extensively investigated. Studies showed (1) an increased risk of CVD in depressed individuals, (2) an even higher risk of depression following acute CVD, and (3) worse prognosis when CVD is complicated by depression^7–10^.

However, the mechanisms underlying this comorbidity remain an issue of investigation. Depression has been shown to aggravate health risks by poor health behaviors (smoking, sedentary behavior, poor diet) which can lead to central obesity, diabetes and CVD^11^. Studies have found that poor health behavior was more common among men than among women^12^. Chronic, low-grade inflammation plays a crucial role for atherosclerosis which underlies CVD^13^. Recently, inflammation has been studied as a potential link between depression and CVD^14^.

Already in the 1990s, meta-analyses^15^ have associated depression with alterations in cellular immunity. A recent meta-analysis^16^ found low-grade inflammation (C-reactive protein (CRP) > 3 mg/l) in 27% of depressed participants (Odds Ratio (OR) = 1.46; compared to healthy controls). Other cross-sectional studies indicated a link between depression symptoms and inflammation (e.g. CRP, interleukin-1 receptor antagonist (IL1-RA), fibrinogen)^17,18^. Lifestyle factors such as smoking and obesity, which are associated with inflammatory responses, accounted for the cross-sectional relationships. A recent meta-analysis, however, found that CRP and interleukin 12 (IL-12) not only had robust associations with depression (irrespective of cofounders such as smoking), but also showed a reduced variability in depressed participants vs. matched controls^19^.

The concept of an inflammatory phenotype of depression^19^ has attracted considerable attention. Chronic and persistent stress not only increases the risk for depression, but may also lead to a loss of the ability to regulate the inflammatory response. Chronically elevated cortisol levels contribute to a vicious cycle of immune dysregulation, hypertension, arteriosclerotic plaques and subsequent CVD. Systemic inflammation may drive depression via pro-inflammatory cytokines. As evidenced by immunotherapy (e. g. IFN–treatment in cancer patients), pro-inflammatory cytokines act on the brain to cause sickness behavior including early-onset somatic symptoms (such as flu-like complaints, pain, fatigue, loss of appetite, decreased motor activity and sleep disturbance), as well as late onset cognitive-emotional symptoms (such as mild cognitive dysfunction including impaired attention and memory, depressed mood, withdrawal, irritability and anxiety)^20^. Sickness behavior is an evolutionary adaptive constellation of changes, however, prolonged inflammatory activation is detrimental to physical and mental well-being^21^.

The cross-sectional findings raise important issues, (1) whether inflammatory mechanisms actually constitute risk factors for the onset of new depression, (2) which immunological markers are relevant, and (3) whether these findings are valid for men and for women.

The meta-analysis by Valkanova et al.^22^ identified a total of eight studies with 14,832 participants investigating the influence of baseline CRP on new onset of depression an average of 5 years later, and three studies (*N* = 3695) examining interleukin 6 (IL-6). Studies were predominantly conducted in the US, with few studies from Great Britain and the Netherlands. Elevated inflammatory markers had a small but statistically significant association with new onset of depression after adjustment for sociodemographic and other (unspecified) risk factors for depression, most importantly baseline depression. In a biobank investigation in a large cohort from Great Britain, Khandaker et al.^8^ established associations of depression with a family history of heart disease, as well as with IL-6, CRP and triglycerides.

Given the limited understanding of the mechanisms underlying the relationship between inflammation and depression, there is no consensus which inflammatory markers are particularly relevant. In their meta-analysis of longitudinal studies, Valkanova et al.^22^ demonstrated stronger effects for CRP vs. IL-6 on depression. By comparison, white blood cell count has been understudied as a marker. Yet, depression could lead to stimulation of hematopoietic cells and reduced vagal nerve activity accelerating the production of white blood cells (WBCs)^23^.

In recent years, awareness of the importance of sex- and gender-sensitive or -specific approaches in medical research has increased^24–26^. Given the substantial epidemiological gender differences in depressive disorders, it seems particularly promising to investigate potentially gender-dependent risk factors for depression. Knowledge about differential vulnerabilities could inform prevention and intervention efforts that are specially geared to the needs of men and women^27,28^.

The role of inflammation in the etiology of depression also needs to be investigated in a gender-specific manner as studies have found greater physiological dysregulation in women (including a host of systems and inflammatory markers), especially after menopause^29^. Experimental evidence supports a gender difference as well: a study found that women reported a stronger decline in their mood after being administered an endotoxin as inflammatory challenge^30^.

However, population-based findings regarding gender-dependent associations of depression and inflammation are scarce and mixed as participants’ outcomes were only seldom analyzed in gender- specific ways. In their large population-based cohort study, Khandaker et al.^8^ reported no gender differences regarding the association of CRP, IL-6 and depression. Surtees et al.^31^ found an association of both depression episode status and history of depression with higher leukocyte counts, but only in men. By contrast, Beydoun et al.^14^ reported that higher total white blood cell count was predictive of an increase of depression symptoms in women, but not in men. In a large Swedish twin study, Kendler et al.^7^ found that the manifestation of CVD was more predictive of the onset of major depression than reverse. However, the temporal pattern differed between men and women: The risk of depression following CVD was much greater for men than for women, particularly in the first year.

### The present study

We used data of a prospective, representative cohort study to address these research gaps. The purpose of this longitudinal study was twofold:

1. to determine whether baseline indicators of inflammation (CRP and WBC) predict new onset of depression at follow-up 5 years later and;
2. to explore differences between men and women with respect to these associations.

In order to identify cases with new onset of depression, we excluded participants with acute depression, a lifetime diagnosis of depression and antidepressant medication at baseline. We controlled sociodemographic factors, socioeconomic status and lifestyle confounders such as smoking and body mass index (BMI) and, importantly, also for baseline depression symptoms.

## Methods

### Procedure and study sample

The Gutenberg Health Study (GHS) is a population-based, prospective, observational single-center cohort study in the Rhine-Main-Region, Germany^32,33^. Its primary aim is to analyze cardiovascular risk factors and their stratification and to foster health prevention in the community. The study protocol and documents were approved by the ethics committee of the Medical Chamber of Rhineland-Palatinate and the local data safety commissioner. All investigations were conducted in line with the Declaration of Helsinki and principles outlined in recommendations for Good Clinical Practice and Good Epidemiological Practice. Participants were included after informed consent. Insufficient knowledge of German and psychological or physical impairment hindering participation led to exclusion. The sample was drawn randomly from the local registry in the city of Mainz and the district of Mainz-Bingen, stratified 1:1 for gender and residence and in equal strata for decades of age. Inclusion criterion was age 35 to 74 years. The response rate (defined as the recruitment efficacy proportion, i.e. the number of persons with participation in or appointment for the baseline examination, divided by the total number of persons with participation in or appointment for the baseline examination, plus those with refusal and those who were not contactable) was 60.3%.

At baseline (assessments between 2007 and 2012), *N* = 15,010 participants were examined. A total of 12,423 of them took part in the follow-up examination (82.8%).

**Figure 1:**
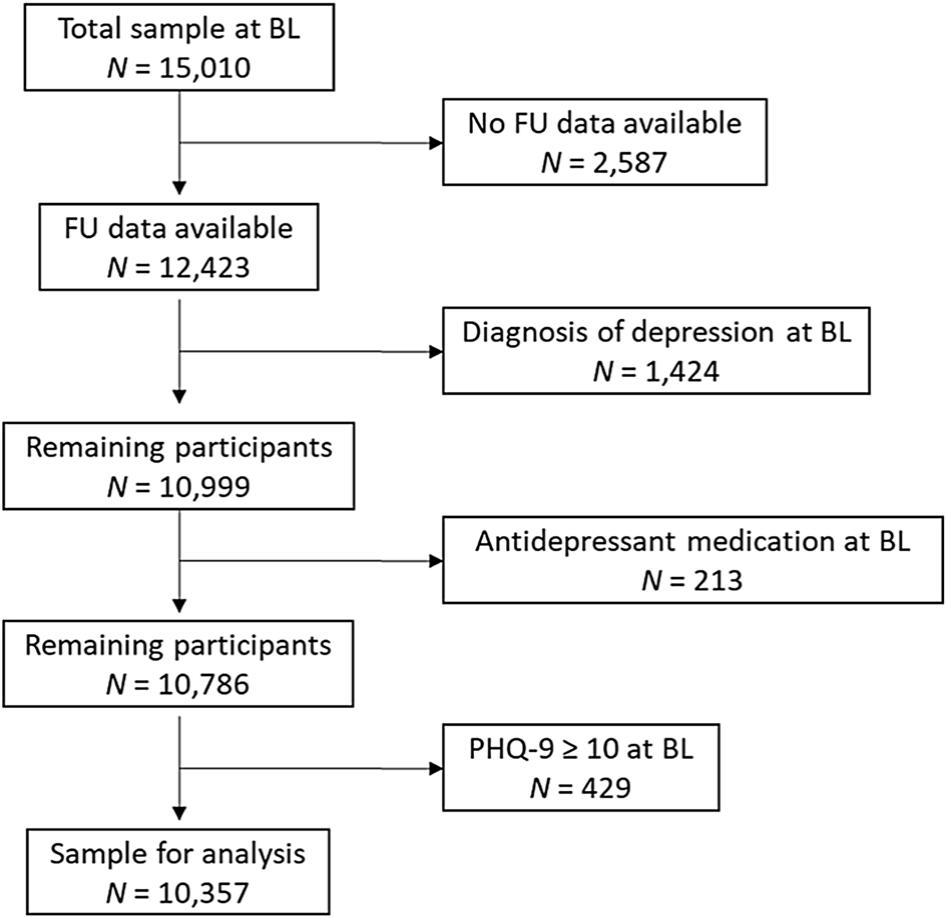
Participant flow. The present work is based on a community sample of *N* = 10,357 participants who took part in assessments at two measurement points and had no evidence for depression at baseline. *BL* baseline, *FU* follow-up.

As the present study concerned new onset of depression, we excluded 2056 individuals with evidence for current depression or history of depression at baseline: 1424 had reported a diagnosis of depression; of the remaining sample, 213 had reported taking antidepressant medication. Lastly, there were 429 participants in the dataset who had surpassed the PHQ-9 cut-off score for relevant symptom burden (≥ 10) at baseline. They were also excluded. Thus, all analyses reported in the following were based on the analyses sample of *N* = 10,357 participants. Figure 1 shows the participant flow.

### Materials and assessment

The 5-h baseline-examination at the study center evaluated the classical cardiovascular risk factors and clinical variables. Participants also underwent a computer-assisted personal interview, and staff performed laboratory examinations from venous blood samples, blood pressure and anthropometric measurements. Examinations were performed according to standard operating procedures by certified medical technical assistants.

### Sociodemographic information

Sociodemographic variables were assessed by self-report: gender, age in years, employment (no/yes), income, living with partner (no/yes), and socioeconomic status (SES). SES was defined according to Lampert et al.^34^. The index combines data based on education, profession and income and ranges from three (lowest socioeconomic status) to 27 (highest socioeconomic status).

### Depression measures

*Depression symptoms* were assessed using the depression module of the Patient Health Questionnaire PHQ-9 (at baseline and follow-up). Participants rated the frequency of occurrence of the nine diagnostic criteria of major depression over the course of the last two weeks. (e.g. “Little interest or pleasure in doing things”) on a Likert scale (0 = not at all, 1 = several days, 2 = more than half the days, 3 = nearly every day). The sum scores ranges from 0 to 27. Presence of relevant symptom burden was defined as a sum score ≥ 10 which has previously shown a sensitivity of 81% and a specificity of 82% for detecting major depressive disorder^35^. Internal consistency was good (Cronbach’s α = 0.80).

*Medication* was registered on site by scanning the bar codes of original packages of drugs taken by participants. Active ingredients were recorded using ATC codes. Three classes of antidepressants were noted: nonselective monoamine reuptake inhibitors (ATC N06AA), selective serotonin reuptake inhibitors (ATC N06AB), and other antidepressants (ATC N06AX).

*Loneliness* was assessed using one item: ’I am frequently alone/have few contacts’ rated from 0 = no, does not apply, to 4 = yes, it applies, and I suffer strongly from it^36^.

### Behavioral measures

Health behavior assessments included *smoking*. Participants’ reports were dichotomized into non-smokers (combining never smokers and ex-smokers) and smokers (combining occasional and frequent smokers).

*Obesity* was defined as a body mass index (BMI) ≥ 30 kg/m^2^.

*Alcohol* consumption was assessed via self-report. Participants reported how often, how many, and which kinds of beverages they consumed (e.g. beer, wine, spirits) in certain quantities. Following a standardized procedure, the number of grams/day was calculated from these responses. Alcohol consumption above the recommended limit was defined as daily consumption ≥ 10 g for women and ≥ 20 g for men (in line with the German threshold for alcohol consumption above tolerance).

*Physical activity* was inquired with the Short Questionnaire to Assess Health-Enhancing Physical Activity (SQUASH; Peters et al.^37^]). The SQUASH captures commuting, leisure time, household, work and school activities with reference to a typical week in recent months. Sleeping, lying, sitting and standing were classified as inactivity^37^. Active sports was presented in quartiles with Q1 denominating the lowest and Q4 the highest quartile of physical activity. Information on days per week, average time per day, and intensity of the activity is used for the calculation of an index score (total minutes of activity multiplied by intensity score, with higher scores reflecting more (intense) activity).

### Interview assessments

During the computer-assisted personal interview, the presence of coronary heart disease was assessed by the question: ‘Were you diagnosed with a stenosis of your coronary vessels?’ Self-reported myocardial infarction (MI), heart failure (HF), stroke, deep vein thrombosis (DVT), pulmonary embolism (PE), and peripheral arterial disease (PAD) were summarized as cardiovascular disease (CVD). Participants were also asked whether they had ever received a definite cancer diagnosis from a physician, and whether they had ever received the definite diagnosis of any depressive disorder (i.e. lifetime diagnosis of depression).

*Diabetes* was defined in individuals with a definite diagnosis of diabetes by a physician or a blood glucose level of ≥ 126 mg/dl in the baseline examination after an overnight fast of at least 8 h or a blood glucose level of > 200 mg/dl after a fasting period of 8 h.

### Laboratory analysis

Venous blood sampling was performed in supine position, and the prior fasting period was documented. C-reactive protein (CRP) concentration was measured in heparin-plasma by a high-sensitivity latex enhanced immunoturbidimetric assay (Abbott Laboratories, Abbott Park, IL). The limit of detection was 0.2 mg/l. Blood cell counts (including white blood cell count (WBC)) were performed on an ADVIA 2120 system (Siemens Healthcare Diagnostics, Eschborn, Germany). Measures of CRP and WBC were done immediately after sampling.

### Statistical analysis

All data underwent quality control by a central data management unit. Data were reviewed for completeness by predefined algorithms and plausibility criteria. Descriptive analyses were performed as absolute and relative proportions for categorical data, means and standard deviations for continuous variables, and median with interquartile range if not fulfilling normal distribution. Inference tests between groups were calculated using t-tests or χ^2^ tests, where appropriate.

In order to identify predictors of incident depression in the analyses sample, we performed logistic regression analyses with depression (PHQ-9 sum score ≥ 10) at follow-up as the criterion variable. The models controlled for depression symptoms at baseline (PHQ-9 sum score), comprised inflammatory markers (CRP, WBC) and their interactions with gender, demographic variables (gender, age, living with a partner, SES) and physical health and health behavior variables (diabetes, cardiovascular disease, cancer, obesity, smoking, physical activity, and excessive consumption of alcohol) as predictors. For analyses, CRP was dichotomized into normal and elevated levels (≥ 3 mg/l, this cut-off value is based on the American Heart Association’s/Centers for Disease Control and Prevention’s guidelines^38^ and previous study procedures^39^). Raw WBCs were substantially left skewed, thus the natural log of WBC was used instead.

In order to determine gender-dependent effects (i.e. following observations of statistically significant interaction terms with gender), we also analyzed women and men separately.

All p-values should be regarded as continuous parameters that reflect the level of statistical evidence, and they are therefore reported exactly. Statistical analysis was carried out using R version 3.6.1.

## Results

In the following, we first report univariate differences between individuals with and without new onset of depression (PHQ-9 sum score ≥ 10) at follow-up (which took place 5 years after the baseline assessment). We then present several logistic regression models predicting depression at follow-up: We tested elevated levels of CRP as a binary predictor (first within the whole sample using an interaction term with gender, secondly in separate analyses in men and women) as well as WBC as a continuous predictor (again both within the whole sample using an interaction term, and separately in men and women).

### Sample characteristics

Table 1 yields an overview of baseline data of the study participants stratified by the presence of clinically relevant depression symptoms (according to the PHQ-9) at follow-up.

**Table 1:** Baseline participant characteristics (stratified by PHQ-9 ≥ 10 at follow-up).

Variable	All (*N* = 10,120)	PHQ-9 ≥ 10 at FU (*N* = 448)	PHQ-9 ≤ 9 at FU (*N* = 9672)	*p*
**Sociodemographic**				
Women (%)	46.1% (4670)	53.1% (238)	45.8% (4432)	**0.003**
Age	54.3 (10.9)	51.2 (10.3)	54.5 (10.9)	** < 0.001**
Living with partner (%)	84.5% (8547)	79.7% (357)	84.7% (8190)	**0.006**
Socioeconomic status	13.45 (4.39)	12.94 (4.26)	13.48 (4.39)	**0.010**
**Inflammation**				
CRP ≥ 3 mg/l (%)	23.9% (2419)	28.0% (125)	23.7% (2292)	**0.041**
WBC (leucocytes/nl)	6.80 (5.75/8.10)	7.10 (5.80/8.66)	6.80 (5.75/8.10)	**0.002**
**Physical health and health behavior**				
Diabetes (%)	7.3% (739)	8.3% (37)	7.3% (702)	0.40
CVD (%)	8.3% (836)	7.9% (35)	8.3% (801)	0.86
Cancer (%)	7.9% (800)	8.9% (40)	7.9% (760)	0.42
Obesity (%)	22.7% (2297)	24.8% (111)	22.6% (2186)	0.30
Smoker (%)	17.2% (1738)	24.3% (108)	16.9% (1630)	** < 0.001**
Physical activity	7260 (5174/9390)	7590 (5500/10,140)	7230 (5156/9360)	**0.019**
Alcohol (% > recom. limit)	28.1% (2836)	22.7% (101)	28.3% (2735)	**0.01**
**Mental distress**				
Loneliness (%)	6.6% (668)	19.0% (85)	6.1% (590)	** < 0.001**
PHQ-9 sum at baseline	3.13 (2.31)	5.48 (2.37)	3.02 (2.25)	** < 0.001**

Bold values indicate statistically significant differences.

Participant characteristics are shown as mean values and standard deviations, medians with interquartile ranges (if not fulfilling normal distribution) or as percentages and absolute numbers.

*CRP* C-reactive protein, *CVD* cardiovascular disease, *FU* follow-up, *PHQ-9* patient health questionnaire-9, *WBC* white blood cell count.

At follow-up, 448 participants (210 men and 238 women) without evidence for depression at baseline reported relevant depression symptoms (4.4% of the overall sample). Group comparisons showed that those participants were more likely to be women, comparatively younger, more likely to live without a partner, and to have a lower socioeconomic status. Participants with new onset of depression were also more likely to smoke, to be physically inactive, and to consume less alcohol at baseline.

More women (5.1%) than men (3.9%) had new onset depression at follow-up. Supplementary Table S1 depicts differences between those with depression at follow-up and those without stratified by gender: Among women, new onset of depression was more common in those who were younger, smoked, and consumed less alcohol. The latter difference was not present among men. By contrast, new onset of depression among men was related to living without a partner, lower socioeconomic status, higher inflammation markers (both CRP and WBC), and being physically inactive.

### Main analyses

We tested associations of CRP and WBC on new onset of depression in separate multivariate logistic regression models.

#### C-reactive protein

Table 2 shows the association of baseline CRP with depression at follow-up.

**Table 2:** Results of the logistic regression analysis: Prediction of new onset of depression at follow-up (based on predictors measured at baseline 5 years earlier, including CRP ≥ 3 mg/l).

Predictors	OR	95% CI	*p*
**Inflammation**			
CRP ≥ 3 mg/l	1.58	1.10; 2.26	**0.013**
Interaction: CRP by gender (women)*	0.55	0.033; 0.91	**0.021**
**Sociodemographic information**			
Gender (women)	1.34	1.02; 1.74	**0.03**
Age	0.98	0.97; 0.99	** < 0.001**
Living with partner	1.14	0.84; 1.55	0.40
Socioeconomic status	0.99	0.96; 1.02	0.06
**Physical health and health behavior**			
Diabetes	1.34	0.84; 2.12	0.22
CVD	1.15	0.75; 1.77	0.53
Cancer	1.38	0.92; 2.07	0.12
Obesity	0.88	0.66; 1.17	0.37
Smoker	1.40	1.07; 1.83	**0.013**
Physical activity	1.00	1.00; 1.00	0.81
Alcohol > recom. limit	0.83	0.64; 1.09	0.18
Loneliness	1.83	1.33; 2.52	** < 0.001**
PHQ-9 sum (baseline)	1.47	1.40; 1.54	** < 0.001**

Bold values indicate statistically significant predictors.

Total model: Nagelkerke *R*^2^ = 0.37.

*CI* confidence interval; *CRP* C-reactive protein; *CVD* cardiovascular disease, *OR* odds ratio, *PHQ-9* patient health questionnaire-9.

*The odds ratio of the interaction term is a ratio of odds ratios (ROR). The OR for CRP > 3 mg/l of 1.58 is to be interpreted as the OR for men with elevated CRP. The OR for women with elevated CRP can be calculated from the ROR and the above OR for men as 1.58 × 0.55 = 0.87.

**Figure 2:**
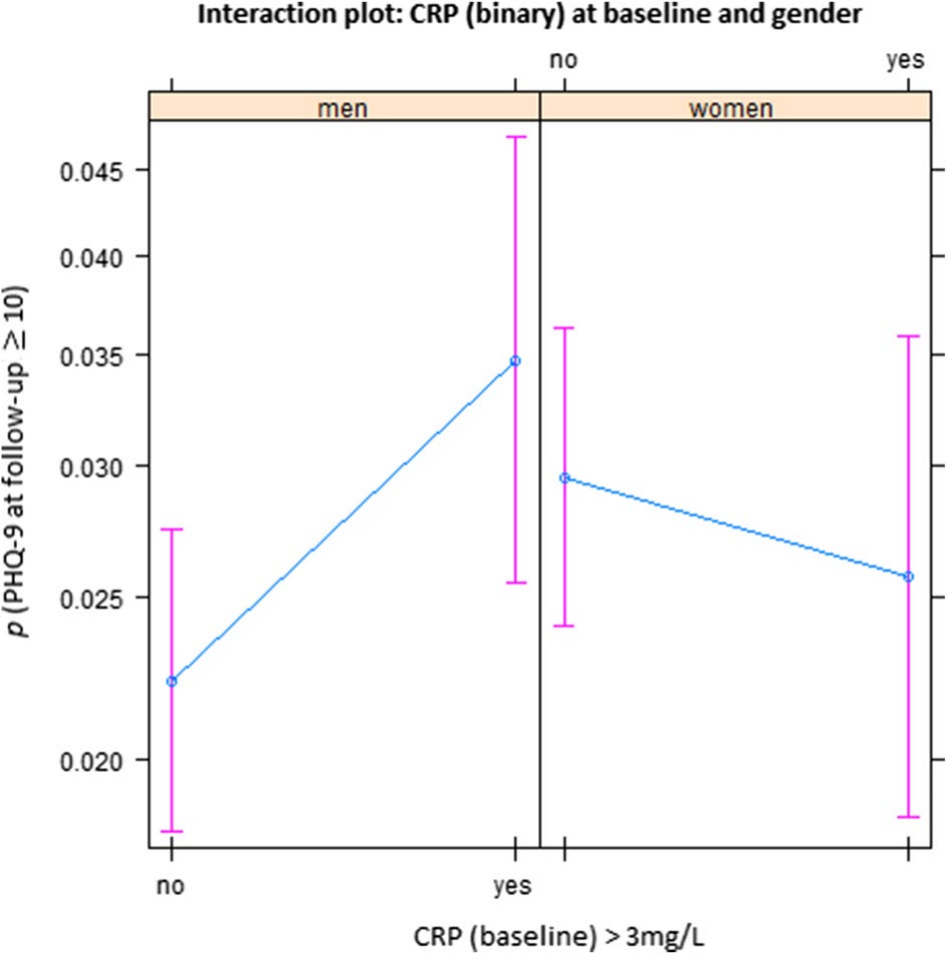
Association with baseline CRP levels and depression symptoms at follow-up in men and women. We observed gender differences regarding the association of elevated levels of CRP at baseline with new onset of depression at follow-up: in men, elevated CRP at baseline was associated with higher PHQ-9 scores at follow-up; whereas in women, no clear effect of baseline CRP on PHQ-9 at follow-up was apparent. The plot results show the probability (*p*) and 95% confidence intervals of depression (PHQ-9 ≥ 10) estimated based on the regression model of the total sample (reported in Table 2), depending on baseline CRP (normal versus elevated) and other predictors held fixed. (Figures were created using R version 3.6.1 https://www.R-project.org/).

Elevated levels of CRP at baseline were predictive of depression at follow-up in the total sample (OR 1.58, 95% CI 1.10–2.26). The interaction term, however, showed that this effect was modified by gender. The interaction of elevated levels of CRP with gender was statistically significant (OR 0.55, 95% CI 0.33–0.91). As illustrated by the interaction plot (Fig. 2), elevated values of CRP predicted new onset depression only in men and not in women. Additional predictors of depression were being a woman, younger age, loneliness, and baseline depression score; there was a trend for lower SES. No significant effects were found for diabetes, cardiovascular disease, cancer, obesity, smoking, physical activity and alcohol consumption.

Table 3 shows separate analyses for men and for women: CRP was predictive of depression (OR 1.4), 95% CI 1.03–2.16) in men only, along with younger age, smoking, loneliness and baseline depression symptoms. In women, lower age, a history of cancer, lower alcohol consumption, loneliness and baseline depression symptoms were predictive of depression, but not CRP.

**Table 3:** Results of the logistic regression analysis: Prediction of new onset of depression at follow-up (based on predictors measured at baseline 5 years earlier, including CRP ≥ 3 mg/l), stratified by gender.

Predictors	Men Nagelkerke *R*^2^ = 0.38			Women Nagelkerke *R*^2^ = 0.37		
	OR	95% CI	*p*	OR	95% CI	*p*
**Inflammation**						
CRP ≥ 3 mg/l	1.49	1.03; 2.16	**0.033**	0.92	0.63; 1.36	0.68
**Sociodemographic**						
Age	0.99	0.97; 1.00	**0.023**	0.97	0.95; 0.99	** < 0.001**
Living with partner	1.29	0.80; 2.10	0.30	1.06	0.71; 1.58	0.79
Socioeconomic status	0.99	0.95; 1.03	0.60	1.00	0.95; 1.04	0.87
**Physical health and health behavior**						
Diabetes	1.54	0.89; 2.67	.12	0.87	0.34; 2.23	0.76
CVD	1.01	0.58; 1.78	0.97	1.21	0.61; 2.42	0.59
Cancer	0.85	0.41; 1.75	0.65	1.82	1.11; 2.99	**0.018**
Obesity	0.98	0.67; 1.44	0.92	0.76	0.48; 1.18	0.22
Smoker	1.53	1.05; 2.22	**0.027**	1.31	0.89; 1.92	0.17
Physical activity	1.00	1.00; 1.00	0.54	1.00	1.00; 1.00	0.85
Alcohol > recom. limit	0.97	0.68; 1.40	0.88	0.67	0.44; 1.00	**0.049**
**Mental distress**						
Loneliness	1.92	1.18; 3.10	**0.008**	1.75	1.14; 2.69	**0.011**
PHQ-9 sum (baseline)	1.56	1.46; 1.67	** < 0.001**	1.38	1.30; 1.48	** < 0.001**

Bold values indicate statistically significant predictors.

*CI* confidence interval, *CRP* C-reactive protein, *CVD* cardiovascular disease, *OR* odds ratio, *PHQ-9* patient health questionnaire-9.

#### White blood cell count

Table 4 presents the association of baseline ln(WBC) with depression at follow-up.

**Table 4:** Results of the logistic regression analysis: Prediction of new onset of depression at follow-up (based on predictors measured at baseline 5 years earlier, including the natural logarithm of WBC).

Predictors	OR	95% CI	*p*
**Inflammation**			
Ln(WBC)	1.88	1.04; 3.42	**0.04**
Interaction: Ln(WBC)* Gender (women)	0.37	0.16; 0.87	**0.023**
**Sociodemographic**			
Gender (women)	7.91	1.48; 42.33	**0.02**
Age	0.98	0.97; 0.99	** < 0.001**
Living with partner	1.13	0.83; 1.54	0.43
Socioeconomic status	0.99	0.97; 1.02	0.65
**Physical health and health behavior**			
Diabetes	1.38	0.87; 2.19	0.17
CVD	1.14	0.74; 1.76	0.55
Cancer	1.39	0.93; 2.09	0.11
Obesity	0.89	0.67; 1.18	0.41
Smoker	1.37	1.04; 1.80	**0.02**
Physical activity	1.00	1.00; 1.00	0.81
Alcohol > recom. limit	0.86	0.66; 1.12	0.25
**Mental distress**			
Loneliness	1.82	1.32; 2.50	** < 0.001**
PHQ-9 sum (baseline)	1.47	1.4; 1.54	** < 0.001**

Bold values indicate statistically significant predictors.

Total model: Nagelkerke *R*^2^ = 0.37.

*CI* confidence interval, *CVD* cardiovascular disease, *OR* odds ratio, *PHQ-9* patient health questionnaire-9, *WBC* white blood cell count.

**Figure 3:**
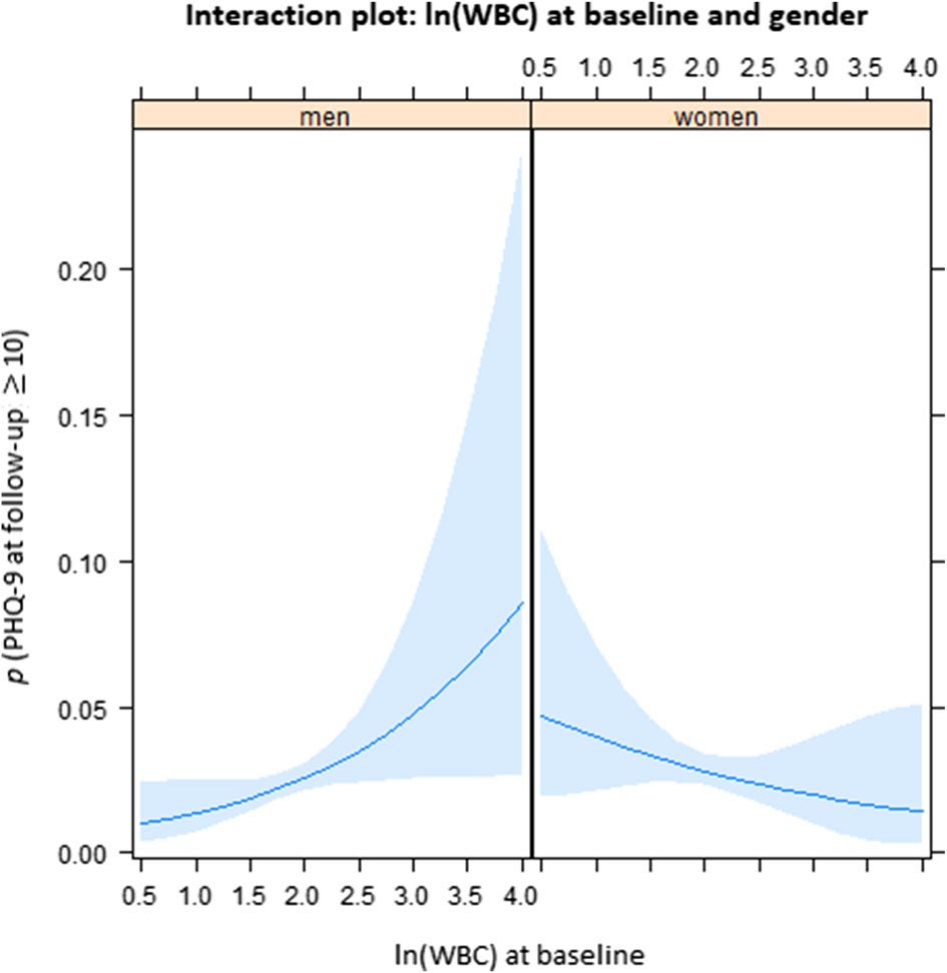
Interaction plot between gender and natural log of white blood cell count as predictors of new onset of depression 5 years later. There were differences between men and women with respect to the association of elevated levels of WBC at baseline with new onset of depression at follow-up: in men, elevated WBC at baseline was more closely associated with higher PHQ-9 sum scores at follow-up. The plot results show the probability (*p*) and 95%-confidence intervals (shaded areas) of depression (PHQ-9), estimated based on the regression model of the total sample (reported in Table 4), depending on baseline natural log of WBC and other predictors held fixed. (Figures were created using R version 3.6.1 https://www.R-project.org/).

As Table 4 shows, ln(WBC) was predictive of depression (OR 1.88, 95% CI 1.04–3.42) in the total sample. Again, the interaction of the inflammation parameter with gender (OR 0.37, 95% CI 0.16–0.87) indicated a stronger increase of depression symptoms based on WBC in men (Fig. 3). Other statistically significant predictors were being a woman, younger age, smoking, loneliness, and baseline depression symptoms. In separate analyses for men and for women (Table 5), these analyses, the association of inflammation and depression in men showed a wide confidence interval, just beyond the conventional level of statistical significance (OR 1.80, 95% CI 0.96–3.37, *p* = 0.07). Risk factors were younger age, loneliness, and baseline depression symptoms. In women, younger age also played a role, and a history of cancer, lower alcohol consumption, loneliness, and baseline depression symptoms.

**Table 5:** Results of the logistic regression analysis: prediction of new onset of depression at follow-up (based on predictors measured at baseline 5 years earlier, including the natural logarithm of WBC), stratified by gender.

Predictors	Men Nagelkerke *R*^2^ = 0.25			Women Nagelkerke *R*^2^ = 0.29		
	OR	95% CI	*p*	OR	95% CI	*p*
**Inflammation**						
Ln(WBC)	1.80	0.96; 3.37	0.07	0.72	0.38; 1.36	0.31
**Sociodemographic**						
Age	0.99	0.97; 1.01	**0.025**	0.97	0.95; 0.99	** < 0.001**
Living with partner	1.27	0.79; 2.07	0.33	1.05	0.70; 1.56	0.82
Socioeconomic status	0.99	0.95; 1.03	0.66	1.00	0.95; 1.04	0.83
**Physical health and health behavior**						
Diabetes	1.58	0.91; 2.72	0.10	0.89	0.34; 2.29	0.80
CVD	1.00	0.57; 1.76	0.99	1.33	0.68; 2.62	0.41
Cancer	0.88	0.43; 1.82	0.73	1.79	1.09; 2.95	**0.021**
Obesity	1.02	0.70; 1.49	0.90	0.75	0.49; 1.15	0.19
Smoker	1.42	0.96; 2.10	0.08	1.36	0.92; 2.01	0.12
Physical activity	1.00	1.00; 1.00	0.51	1.00	1.00; 1.00	0.83
Alcohol > recom. limit	1.02	0.71; 1.46	0.93	0.66	0.44; 1.00	**0.047**
**Mental distress**						
Loneliness	1.89	1.17; 3.05	**0.01**	1.75	1.14; 2.69	**0.011**
PHQ-9 sum (baseline)	1.56	1.45; 1.67	** < 0.001**	1.38	1.30; 1.48	** < 0.001**

Bold values indicate statistically significant predictors.

*CVD* cardiovascular disease, *PHQ-9* patient health questionnaire-9, *WBC* white blood cell count.

## Discussion

The present study investigated the longitudinal associations of inflammatory markers and new onset of depression symptoms in a large and representative German community sample. We had previously excluded all cases of acute depression, previous diagnosis of depression, and those on antidepressants at baseline in order to identify cases with new onset of depression at follow-up.

Based on two markers, CRP and WBC, we found inflammation at baseline predictive of new cases of depression at follow-up 5 years later. The effects of both markers were independent of depression symptoms at baseline. Increased CRP levels raised the risk of subsequent depression by 1.58 (95% CI 1.010 to 2.16) and ln WBC by 1.88 (95% CI 1.04 to 3.42), even when adjusting for sociodemographic, physical health, health behavior variables, and baseline depression symptoms.

Unlike previous studies, we used interaction terms with gender in our models and also conducted separate analyses for men and women. We found significant interactions of CRP and WBC with gender. In separate analyses, CRP was only predictive of new onset of depression in men, but not in women. These findings compliment previous research showing a stronger increase of CRP in depressed men vs. women^40,41^. Additional factors were lower age, smoking, loneliness and baseline depression symptoms. In women, lower age, a history of cancer, lower alcohol consumption, loneliness and baseline depression symptoms were predictive of depression, but not CRP. As consumption of alcohol is quite widespread in Germany, especially in social contexts, and as the recommended limit (for women) is reached with few drinks, the negative predictive valence of alcohol consumption might have been related to little social contact (and related social drinking) which aggravates the risk for depression^42^.

Regarding WBC, we found a trend for men (OR 1.80; 95% CI 0.96 to 3.37 per natural log-unit increase), along with loneliness, and baseline depression symptoms. These patterns of predictors corroborate previous findings of large cohort studies^43^.

In our study, inflammation increased the risk of incident depression in men. Especially with respect to CRP, it was suggested that the relationship with depression symptoms only applies to men^44,45^. A possible explanation is provided by the hypothesis that inflammation arises from the dysregulation of hormonal systems. In this context, estrogen might have beneficial effects (which are also indicated by the positive influence of hormone replacement therapy on women’s mood^46^), for instance due to its anti-inflammatory properties^47,48^.

What is more, there are differences in the basic immune response of men and women^49^ that could shape the association between inflammation markers and depression symptoms. Women’s response to infection is more pronounced; this pertained to the overwhelming majority of characteristics of immune components including both the innate and the adaptive immune system^50^. Although this response pattern has been deemed adaptive as women have a better prognosis after experiencing e. g. sepsis^51^, it might have downsides as they are more likely to suffer from autoimmune diseases than men^52^.

The exact mechanisms underlying the present findings, however, remain to be elucidated, as both inflammation and depression could be consequences of an underlying factor like allostatic load. Therefore, further characterization of an inflamed depression subtype could be a worthwhile pursuit. However, the gender difference and the high variability of the effect of inflammation on depression in men suggest that inflammation is not a necessary cause of depression. Instead, the results indicate gender-specific pathways in the development of depressive disorders. Regarding the generally more pronounced vulnerability for internalizing mental disorders, studies have shown that these disparities co-occur with the onset of puberty^2^. It has been discussed that differential risk depends on hormonal changes and societal gender role concepts alike (which become more salient around this time)^2^.

Within the present study, we observed that older age protected against new onset of depression. At baseline participants were between 35 and 74 years old. Correspondingly, the prevalence of major depression decreased with age in previous community studies^53^. Along these lines, it is important to note that hormonal changes over the course of a persons’ lifespan shape physiological regulation including inflammatory processes^29^. Moreover, studies have found that the gender gap in depression narrows after the fertile periods of women’s lives whereas women’s susceptibility to chronic inflammatory diseases approaches men’s as they age^54^. In a previous meta-analysis, depression in older adults was more likely to co-occur with physical complaints (including e.g. gastrointestinal symptoms) than in the young^55^.

As inflammation is a core mechanism of cardiovascular, metabolic and other chronic somatic diseases, the present results help to understand the high prevalence of somatic comorbidity in depression. In our investigation, presence of CVD was not associated with depression symptoms at follow-up (in models that controlled for inflammatory markers and lifestyle factors such as including bodyweight, physical activity, smoking, and alcohol consumption). Previous studies have found that depression was a risk factor for CVD^56,57^. Additionally, an earlier prospective cohort study showed that depression symptoms predicted health risk behaviors five years later, indicating a potential mediator^11^ of the relationship between depression and CVD. It is important to note that an individual’s lifestyle constitutes a modifiable risk factor that can be addressed by population-based intervention and prevention programs (e. g. smoking cessation; physical exercise). Beneficial effects of lifestyle changes on inflammation were recently reported^58^ and could contribute to recovery from depression symptoms, too. Other examples of interventions to reduce depression and CVD address nutrition (e.g. by incorporating anti-inflammatory foods and supplements^23^) and the reduction of chronic stress as a major driver of inflammation (e. g. by psychotherapy, online interventions, relaxation exercises).

### Strengths and limitations

We report findings from a sizeable community cohort with equal proportions of men and women. As previously recommended^19^, we excluded participants with any indication of previous depression (based on baseline assessment, lifetime diagnosis, and intake of antidepressants). We also controlled for lifestyle variables associated with depression and with inflammation (smoking, obesity) and, importantly, for baseline depression symptoms.

In addition to CRP, one of the most frequently studied and robust indicators of inflammation, we included WBC to expand on previous research. Yet, we are aware that we used only two inflammatory markers, and other acute-phase markers (e. g. fibrinogen) or other soluble immune markers (e. g. cytokines such as IL-6) were not included. Given the fact that they were the focus of a large body of previous research concerning the etiology and/or maintenance of depression^22^, this limits the comparability of our investigation with previous studies. In addition, the combined study of a wider range of inflammatory markers could elicit different (gender-specific) pathogenic pathways.

Our primary outcome measure was the PHQ-9. It is one of the most-widely used self-report questionnaires to assess depression symptoms. While we inquired previous diagnoses of depression, we did not ascertain medical diagnoses of depression from medical files. Given the limited period of two weeks captured by the PHQ-9, we cannot preclude that participants were depressed at other times before the follow-up assessment took place. While we used all information available to exclude individuals who had previously been depressed, an unknown proportion of included participants might have fulfilled the criteria of depression in the past without proper diagnosis. Also, they might not have disclosed previous diagnoses. Other potential predictors such as family history of depression were not assessed. Further, alcohol consumption exceeding the recommended limit was defined based on self-reports that could not be objectively validated. We also assessed previous diagnosis of CVD and cancer via self-reported diagnoses. We did not differentiate for time since diagnosis or (in the case of cancer) stage and treatment status, which presents a limitation.

## Supplementary Information

Supplementary Information.

## Data Availability

The written informed consent of the study participants is not suitable for public access to the data and this concept was not approved by the local data protection officer and ethics committee. Access to data at the local database in accordance with the ethics vote is offered upon request at any time. Interested researchers make their requests to the Principal Investigator of the GHS (Philipp.Wild@unimedizin-mainz.de).
