# Supplementary material for: Inflammation predicts new onset of depression in men, but not in women within a prospective, representative community cohort
Source: Sci Rep. 2021 Jan 26;11:2271. doi: 10.1038/s41598-021-81927-9 (PMC7838404; doi:10.1038/s41598-021-81927-9)
Supplement: Supplementary file 1 — Supplementary Information. [file 41598_2021_81927_MOESM1_ESM.pdf]

Supplement for “Inflammation predicts new onset of depression in men, but not in women within a prospective, representative community cohort”

Authors: Mareike Ernst, Elmar Brähler, Daniëlle Otten, Antonia M. Werner, Ana N. Tibubos, Iris Reiner, Felix Wicke, Jörg Wiltink, Matthias Michal, Markus Nagler, Thomas Münzel, Philipp S. Wild, Jochem König, Norbert Pfeiffer, Andreas Borta, Karl J. Lackner, Manfred E. Beutel

Supplementary Table 1. Baseline participant characteristics (stratified by PHQ-9≥10 at follow-up) by gender

	Men				Women			
Variable	All (*N*=5,450)	PHQ-9≥10 at FU (*N*=210)	PHQ-9≤9 at FU (*N*=5,240)	*p*	All (*N*=4,670)	PHQ-9≥10 at FU (*N*=238)	PHQ-9≤9 at FU (*N*=4,432)	*p*
Inflammation								
CRP >= 3 mg/l (%)	21.6% (1,177)	29.0% (61)	21.3% (1,116)	**.010**	26.6% (1,240)	27.0% (64)	26.6% (1,176)	.88
WBC	6.73 (5.69/8.01)	7.20 (6.08/8.97)	6.71 (5.68/7.99)	**<.001**	6.90 (5.84/8.20)	7.09 (5.70/8.55)	6.90 (5.85/8.18)	.42
Sociodemographic								
Age	54.6 (11.0)	52.5 (10.8)	54.7 (11.0)	**.005**	54.0 (10.9)	50.1 (9.8)	54.2 (10.9)	**<.001**
Living with partner (%)	87.0% (4,742)	81.0% (170)	87.3% (4,572)	**.012**	81.5% (3,805)	78.6% (187)	81.7% (3,618)	.23
Socioeconomic status	14.15 (4.48)	13.42 (4.42)	14.18 (4.48)	**.017**	12.64 (4.13)	12.52 (4.09)	12.64 (4.13)	.66
Physical health and health behavior								
Diabetes (%)	9.2% (499)	12.9% (27)	9.0% (472)	.066	5.1% (240)	4.2% (10)	5.2% (230)	.65
CVD (%)	10.4% (561)	11.7% (24)	10.3% (537)	.56	5.9% (275)	4.6% (11)	6.0% (264)	.48
Cancer (%)	7.2% (390)	6.2% (13)	7.2% (377)	.68	8.8% (410)	11.3% (27)	8.6% (383)	.16
Obesity (%)	24.3% (1,325)	28.6% (60)	24.2% (1,266)	.16	20.8% (973)	21.4% (51)	20.8% (973)	.81
Smoker (%)	18.0% (981)	27.5% (57)	17.6% (924)	**<.001**	16.2% (757)	21.4% (51)	15.9% (706)	**.030**
Physical activity	7,350.00 (5,031.67)	7,665.00 (5,476.67)	7,320.00 (4,995.00)	**.035**	7,499.96 (3,438.69)	7,906.16 (3,845.06)	7,478.01 (3,414.59)	.13

Alcohol (% > recom. limit)	30.7% (1,669)	29.0% (60)	30.7% (1,609)	.76	25.0% (1,167)	17.2% (41)	25.4% (1,126)	**.007**
Mental distress								
Loneliness (%)	5.6% (300)	17.4% (36)	5.1% (264)	**<.001**	7.9% (366)	20.2% (47)	7.3% (319)	**<.001**
PHQ-9 sum at baseline	2.89 (2.27)	5.58 (2.40)	2.79 (2.20)	**<.001**	3.41 (2.31)	5.39 (2.34)	3.31 (2.26)	**<.001**

Note. Participant characteristics are shown as mean values and standard deviations, medians with interquartile ranges (if not fulfilling normal distribution) or as percentages and absolute numbers. Abbreviations: CRP=C-reactive protein; CVD=Cardiovascular disease; FU=follow-up; PHQ-9=Patient Health Questionnaire-9; WBC=White blood cell count.
